# Early efficacy observation of the unilateral biportal endoscopic technique in the treatment of multi-level lumbar spinal stenosis

**DOI:** 10.1186/s13018-024-04575-5

**Published:** 2024-02-03

**Authors:** Dingding Jia, Xin Qiao, Xuepan Wang, Shaoqing Li, Qiang Li, Yunbing Hao, Xiangping Peng

**Affiliations:** Department of Orthopedic Surgery, Orthopedic Hospital of Xingtai, 202 Bayi Street, Xingtai, 054000 China

**Keywords:** Unilateral biportal endoscopy, Design of incisions, Multi-level lumbar spinal stenosis, Curative effect

## Abstract

**Background:**

To explore the early curative effect of unilateral biportal endoscopy (UBE) in the treatment of multi-level lumbar spinal stenosis with the help of multiple small incisions.

**Methods:**

A retrospective analysis was performed on 26 patients with multi-level lumbar spinal stenosis treated by UBE in our hospital from August 1, 2021, to March 1, 2022. We collect patients’ basic medical records and independently design surgical incisions. The visual analog score (VAS) and Oswestry Disability Index (ODI) were compared before surgery, 7 days after surgery and 6 months after surgery. Spinal canal diameters on CT were compared before surgery and 7 days after surgery. The modified MacNab standard was used to evaluate the efficacy satisfaction at 6 months after operation.

**Results:**

In this study, 26 patients were operated according to the predetermined surgical plan. The operative time was 145 ± 40.11 min, the intraoperative blood loss was 156.25 ± 44.32 ml, and the postoperative hospital stay was 4.79 ± 1.31 days. The VAS scores of postoperative lumbago and leg pain were lower than those before surgery (*P* < 0.05). The postoperative ODI score was significantly different from that before surgery (*P* < 0.05). The postoperative CT sagittal diameter was significantly different from that before surgery (*P* < 0.05). The curative effect of modified MacNab was 76.92% when followed up 7 days after surgery. The curative effect of modified MacNab was 92.31% when followed up 6 months after surgery, which was significantly improved compared with 7 days after surgery.

**Conclusion:**

Under multiple small incision channels, UBE can effectively treat multi-level lumbar spinal stenosis, significantly relieve the clinical symptoms of patients, and significantly improve the quality of life of patients. It is a safe and feasible minimally invasive surgical treatment method for multi-level lumbar spinal stenosis.

## Background

Lumbar spinal stenosis is one of the most common degenerative diseases of the spine, requiring surgical intervention for patients with severe or progressive nerve damage, persistent pain, and failure to respond to non-surgical treatment [1]. Traditional open surgery is a common way to treat degenerative diseases of the lumbar spine, including open decompressive laminectomy, foraminotomy and fusion. But its large iatrogenic trauma has a negative impact on the muscles and ligaments and other anatomical tissues, resulting in muscle atrophy, lumbar instability, long-term back pain, etc. [2].

Ogden et al. [3] introduced less invasive surgical techniques such as bilateral decompression with a unilateral approach have proven effective in the literature. In patients with LSS, especially geriatric patients, bilateral decompression with a unilateral approach (BDUA) could result in less intraoperative blood loss and a short stay in hospital and it could be quite effective in reducing pain, improving walking distance and quality of life without producing significant side-effects. Moreover, there was no worsening of the grade or degree of slipping in patients with degenerative spondylolisthesis. This technique then evolved into microendoscopic decompression with the use of microscopy and percutaneous foraminal endoscopy. In 1996, De Antoni et al. [4] reported the unilateral biportal endoscopy (UBE) technique for the first time. By using two independent channels of observation and operation, the unilateral biportal endoscopy has a large operation space and a wider selection of instruments. The operation flexibility and work efficiency were significantly improved. UBE preserves the back muscle, has smaller incisions, less intraoperative blood loss, less postoperative back pain, and shorter hospital stay [5–9].

With the development of technology, the application of UBE in spinal surgery has been gradually expanded [10]. Currently, more reports on UBE focus on the treatment of single-level degenerative diseases of the lumbar spine, while the therapeutic effect of multi-level lumbar spinal stenosis is rarely reported [11]. Therefore, in this study, we aim to analyzed the early curative effect of unilateral biportal endoscopy (UBE) in the treatment of multi-level lumbar spinal stenosis.

## Materials and methods

### General information

Inclusion criteria: ① Lumbar spinal stenosis at more than 2 levels (including 2 levels); ② It has obvious symptoms of nerve compression, which is ineffective after formal conservative treatment and requires surgical treatment. Exclusion criteria: ① Lumbar instability, lumbar vertebra spondylolisthesis II degree or above; ② Single level lumbar spinal stenosis; ③ spinal fracture; ④ Past history of lumbar surgery and spinal infection; ⑤ Patients with neoplastic diseases or other patients who cannot tolerate surgery. A total of 200 patients underwent surgery for lumbar spinal stenosis in Xingtai Orthopedic Hospital from August 1, 2021, to March 1, 2022. 26 patients were selected for UBE surgery according to the inclusion and exclusion criteria, including 16 males and 10 females, aged from 35 to 70 years old, with an average age of 51.22 ± 8.19 years old, Body mass index 24.36 ± 2.46 kg/m^2^. Multilevel lumbar spinal stenosis (including 2 levels): L2/L3 and L3/L4:4 cases. L3/L4, L4/L5:8 cases; L4/L5, L5/S1:12 cases; L3/L4, L4/L5, L5/S1:2 cases. All enrolled patients signed informed consent and were approved by the Ethics Committee of Xingtai Orthopedic Hospital. The operations were performed by the same group of doctors.

### Surgical methods

**Fig. 1:**
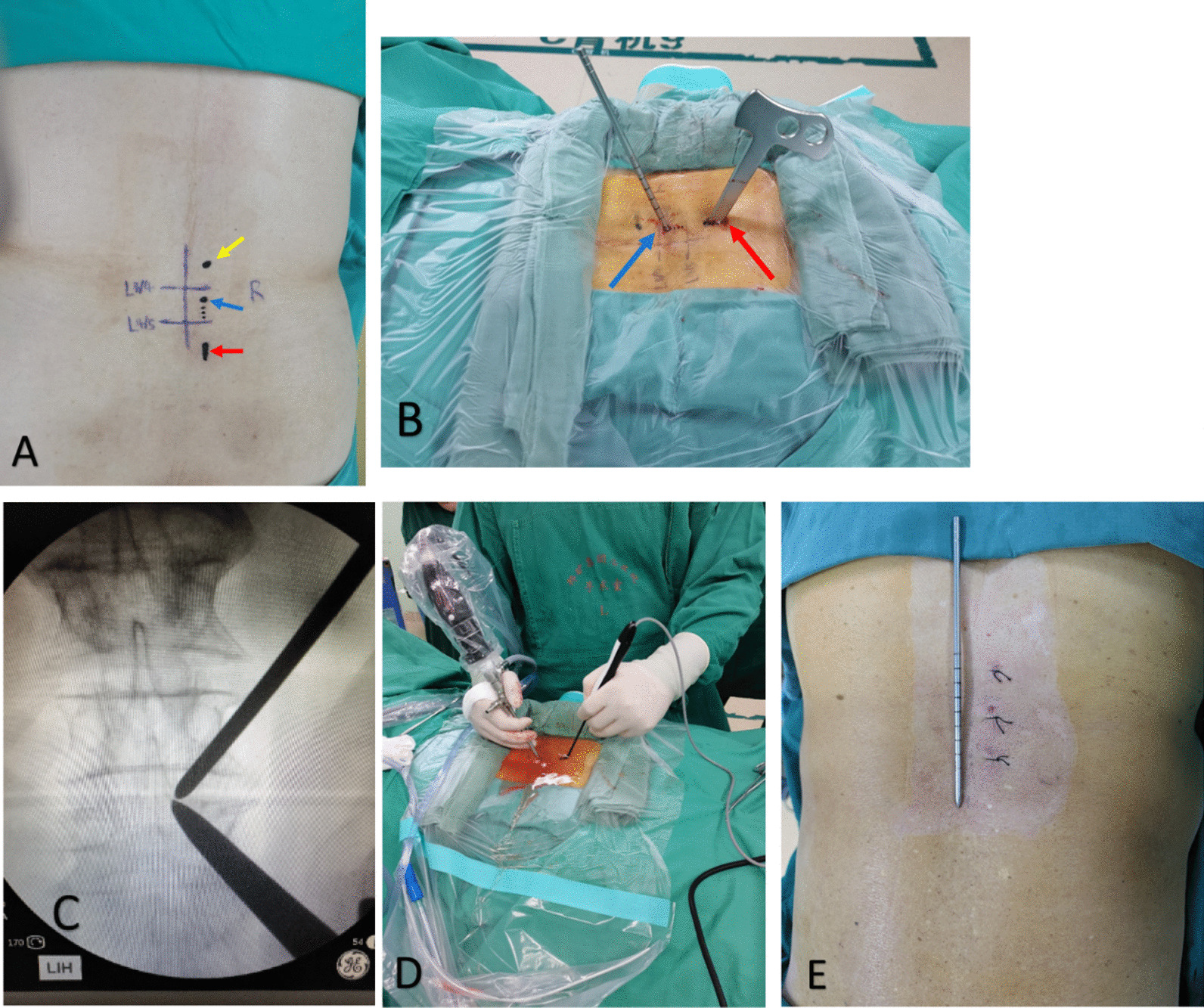
The red arrow in (**A**) indicates the operation channel in the decompression L4/L5 segment, and the blue arrow indicates the observation channel in this segment. When L3/L4 was decompressed, the L4/L5 observation channel (blue arrow) was cut along the dotted line and served as the operation channel. The yellow arrows show the viewing channels at decompression L3/L4. Body surface location diagram in (**B**), decompression is carried out from tail end to head end, red is the operation channel, blue is the observation channel. **C** shows the positioning map under perspective. **D** shows the intraoperative drawings. **E** shows the general incision after operation

Taking the right surgical approach at L3–L5 as an example, the patient lies prone on the operating table after successful general anesthesia, and the surgical segment decompression sequence is carried out from the working channel end to the observation channel end. Taking the L3–L5 segment as an example, the L4/L5 segment decompression is performed first, and the observation channel at the head end is extended appropriately after the decompression is completed. It will be used as a working channel for L3/L4 segment decompression to complete the decompression of this segment. Specific steps: Under fluoroscopy, the lower edge of the root of the L3 and L4 spinous processes and the points of the vertebral lamina were located, and the body surface projection of the right L3, L4 and L5 pedicles was made and marked. Routine disinfection was performed, and U-shaped water outlet was made. The lower edge of the L4–5 spinous process and the laminae were 1.5 cm from each side of the marks at the junction of the L4–5 spinous process and the medial edge of the pedicle as the center point. Incisions were made about 0.5 cm (cephalic, observation channel) and 1 cm (caudal, operation channel) (Fig. 1). A muscle stripper was used to peel off the muscle in the operating channel, and a stepwise dilator was used to dilate the muscle. An endoscope was inserted into the observation channel. Under endoscopic supervision, the lower part of the right L4 lamina and the upper part of the L5 lamina were removed in the operating channel, the medial part of the upper and lower articular processes were removed, the insertion of the yellow ligament was fully dissociated, the right yellow ligament was removed, the dural sac was exposed, and the right L5 nerve root was decompressed. The root of the L4 spinous process was removed by grinding drill, and the medial bone plate of the left L4 lamina was removed, the left L4–L5 yellow ligament was excised, the left dural sac was exposed, and the L5 nerve root was decompressed. After completed decompression, the incision of the observation channel at the head was extended appropriately as the operation channel for decompression at the L3/4 level. The L3–4 level was treated in the same way, the dural sac and bilateral L4 and L5 nerve roots were investigated for relaxation without compression, plasma radiofrequency electroknife was used to stop bleeding thoroughly, and the incisions were sutured with 2–0 Mousse line.

### Postoperative management

**Fig. 2:**
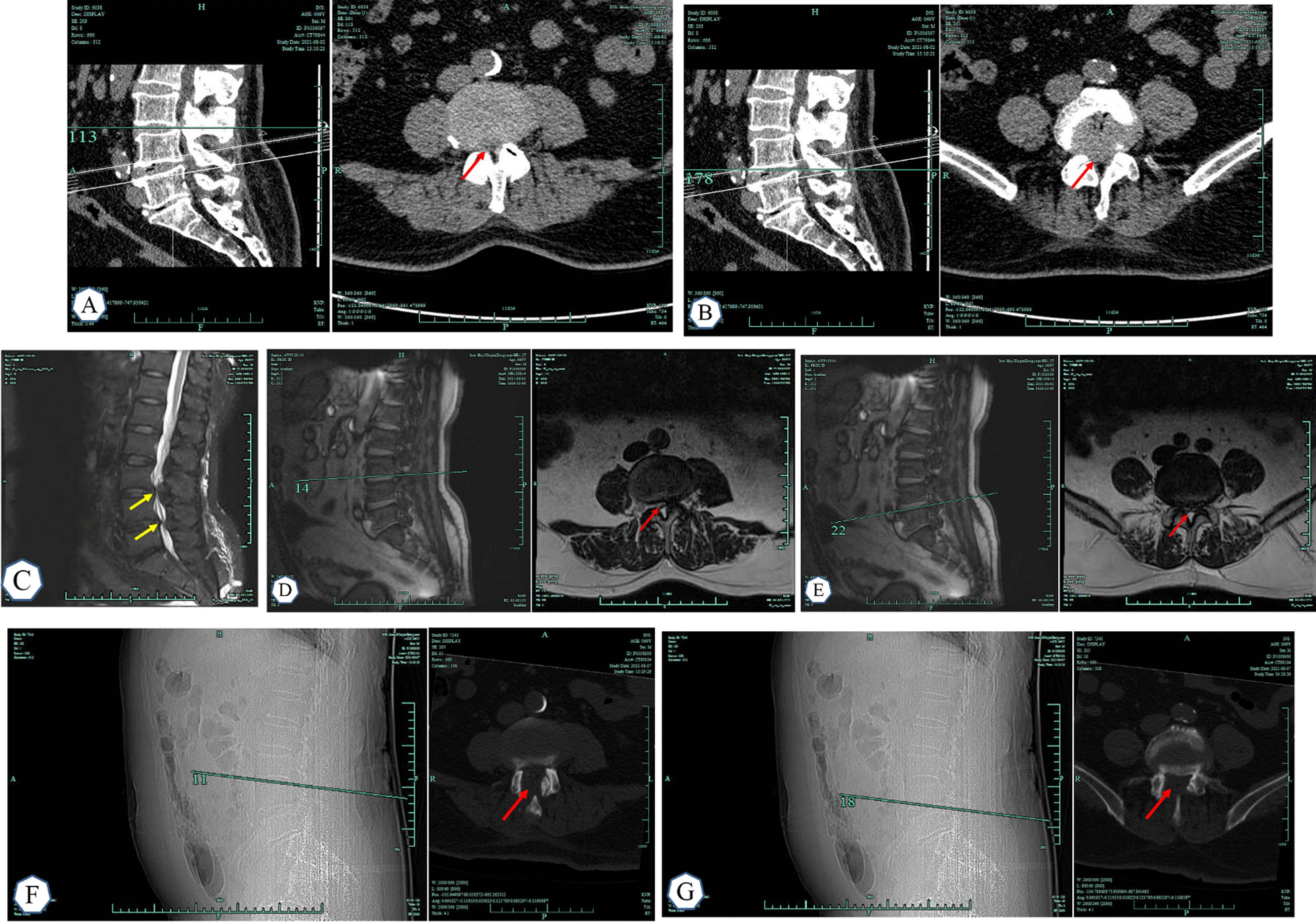
Typical case **A**–**E** showed CT and MRI examinations of the lumbar spine before surgery. Arrows showed L3–L5 lumbar spinal stenosis area.The arrows in (**F**) and (**G**) show the decompression area during the postoperative CT examination

On the first day after surgery, straight leg elevation exercises were performed to prevent nerve root adhesion, lumbar and back muscle functional exercises were performed, and lumbar CT was reviewed. On the second day after surgery，pathents wear thoracolumbosacral orthosis brace to get out of bed and perform adaptive walking function exercise (Fig. 2 Typical case).

### Observation index

The basic data of patients were collected, including age, sex, body mass index, surgical segment, surgical bleeding, surgical duration, postoperative hospital stay (days), and complications. VAS scores and ODI were compared before surgery, 7 days and 6 months after surgery. Spinal canal diameters on CT were compared before surgery and 7 days after surgery. The patients' satisfaction with postoperative efficacy was evaluated by modified MacNab 6 months after surgery. The modified MacNab evaluation criteria divided the postoperative efficacy of patients into four grades: excellent (complete disappearance of symptoms, return to the original work and life), good (mild symptoms, mild limited activities, no impact on work and life), acceptability (symptoms reduced, limited activities, affect normal work and life), poor (no difference before and after treatment, or even worse).

### Statistical analysis

SPSS 20.0 (Statistical Product and Service Solutions 20.0, Chicago, IL) was used for statistical analysis. Quantitative data are expressed as mean ± standard deviation. One-way analysis of variance (ANOVA) was used to compare VAS score and ODI score before surgery, 7 days and 6 months after surgery, and LSD (Least—SignificantDifference) T test was performed.*χ*^2^ test was used to analyze the classified data, and Mann–Whitney test was used to analyze the continuous data that did not conform to the normal distribution. *P* < 0.05 was considered statistically significant.

## Results

In this study, 26 patients underwent surgery smoothly, and all enrolled patients underwent surgery according to the scheduled surgical plan. The operative time was 145 ± 40.11 min, and the intraoperative blood loss was 156.25 ± 44.32 ml. Postoperative hospitalization time was 4.79 ± 1.31d. One case of intraoperative rupture of the spinal dural was treated with gelatin sponge. The incision healed in one stage and the symptoms recovered satisfactorily. Subcutaneous hematoma was treated in 1 case. After sterilizing, 60 ml of light red liquid was extracted with a syringe and then wrapped with pressure. The patient recovered well (Table 1).

**Table 1:** Basic data of 26 patients

Basic index	Statistical magnitude
Age (years)	51.22 ± 8.19
Gender (*n*)	
Male	16
Female	10
Body mass index (kg/m^2^)	24.36 ± 2.46
Operative segment (*n*)	
L2/L3 + L3/L4	4
L3/L4 + L4/L5	8
L4/L5 + L5/S1	12
L3/L4, L4/L5, L5/S1	2
Intraoperative hemorrhage (ml)	156.25 ± 44.32
Operation time (min)	145 ± 40.11
Postoperative hospital stay (Days)	4.79 ± 1.31
Complication (n)	2

All patients were followed up for 6 months. The preoperative leg pain VAS score was 7.58 ± 1.42, and the postoperative leg pain score was 1.77 ± 0.82, which was significantly lower than that before surgery (*t* = 18.80, *P* < 0.001). The postoperative leg pain score was 0.65 ± 0.54, which was further significantly lower than that before surgery (*t* = 22.62, *P* < 0.001). The lumbago VAS score before surgery was 4.65 ± 1.01, and the lumbago VAS score for 7 days after surgery was 2.23 ± 0.59, which was significantly lower than that before surgery (*t* = 12.04, *P* < 0.001). The lumbago VAS score 6 months after surgery was 0.59 ± 0.58, which was significantly lower than that before surgery (*t* = 15.70, *P* < 0.001). ODI score was 71.00 ± 12.79 before surgery and 24.08 ± 2.50 for 7 days after surgery, which was improved compared with that before surgery (*t* = 17.68, *P* < 0.001). The ODI score at 6 months after operation was 13.69 ± 2.17, which was significantly improved compared with that before operation (*t* = 26.33, *P* < 0.001). Spinal canal diameters on CT were 0.30 ± 0.84 cm before surgery and 0.85 ± 0.69 cm for 7 days after surgery, which was improved compared with that before surgery (*t* = − 45.51, *P* < 0.05) (Table 2). At the 7-day postoperative follow-up, the curative effect of modified MacNab was excellent: 8 (30.77%), good 12 (46.15%), acceptable 4 (15.38%), poor 2 (7.69%), the rate of excellent and good reached 76.92%. At the follow-up 6 months after the operation, the curative effect of modified MacNab was excellent 10 (38.46%), good 14 (53.85%), acceptable 2 (7.69%), poor 0 (0.00%), and the rate of excellent and good reached 92.31%, which was significantly improved compared with 7 days after the operation (Table 3).

**Table 2:** Comparison of preoperative and postoperative clinical evaluation indexes

Evaluation time	Number of cases	Leg pain VAS score	Lower back pain VAS score	ODI score (%)	Diameter (cm)
Preoperative (1)	26	7.58 ± 1.42	4.65 ± 1.01	71.00 ± 12.79	0.30 ± 0.84
7 Days after surgery (2)	26	1.77 ± 0.82	2.23 ± 0.59	24.08 ± 2.50	0.85 ± 0.69
6 Months after surgery (3)	26	0.65 ± 0.54	0.59 ± 0.58	13.69 ± 2.17	

Preoperative and postoperative comparison		*T*	*P*	*T*	*P*	*T*	*P*	*T*	*P*
(1): (2)		18.80	< 0.001*	12.04	< 0.001*	17.68	< 0.001*	− 45.51	< 0.05*
(1): (3)		22.62	< 0.001*	15.70	< 0.001*	26.33	< 0.001*		

*VAS* visual analog score, *ODI* Oswestry Disability Index

*Indicates statistical difference, *P* < 0.05

**Table 3:** Postoperative evaluation results of modified MacNab

Postoperative follow-up	Efficacy evaluation (%)			
	Excellent	Good	Acceptability	Poor
7 Days after surgery	8 (30.77%)	12 (46.15%)	4 (15.38%)	2 (7.69%)
6 Months after surgery	10 (38.46%)	14 (53.85%)	2 (7.69%)	0 (0.00%)

## Discussion

The purpose of surgical treatment of lumbar spinal stenosis is to perform thorough decompression of the “responsible segment,” effectively release the compressed spinal cord and nerve roots, and at the same time reduce the damage to the spinal stability as much as possible, so as to effectively relieve the patient’s symptoms. Total laminectomy is the most common surgical procedure in clinical practice, which has the advantages of broad operating space, clear visual field and full decompression, but it also has the disadvantages of great damage to the spinal structure, long operation time, and prone to muscle weakness, muscle atrophy, lumbar instability or spondylolisthesis in the later period [12]. In addition, with the increasing problem of aging society, the incidence trend of lumbar spinal stenosis increases, and most patients mostly have varying degrees of osteoporosis, which will reduce the screw control force, resulting in screw loosening, falling off, fusion cage sinking, intervertebral space is not fused to form pseudarthrosis and other complications. Moreover, iatrogenic spinal instability caused by open surgery cannot be avoided [13], so the selection of surgical methods for the treatment of lumbar spinal stenosis should follow the principles of minimally invasive, simple and precise as much as possible.

The UBE combines the advantages of microscopy and endoscopy. UBE technology has two channels, one of which provides surgical field of view and continuous water perfusion, and the other channel is used for instrument operation. Therefore, UBE has an independent visual operating field. A separate operating channel increases the mobile range of surgery, making operation easier and also provides a good visual field in the contralateral foraminal area [14]. Therefore, compared with single-channel endoscopic technology, UBE can provide a broader field of vision and larger operating space, so as to achieve faster and more adequate decompression [15]. We have found in practice that even in obese patients, the difficulty of surgery is not significantly increased because the access limits the movement of instruments less. In our study, we found that avoiding excessive muscle stripping while setting up the working passage can also reduce the amount of blood loss. In this study, 26 patients with multi-level lumbar spinal stenosis did not increase the amount of blood loss, and the operation time was within the forecast. The curative effect reached the expected effect for 7 days after surgery and 6 months after surgery.

Based on the summary of the treatment of multi-level lumbar spinal stenosis, we believe that the selection of the sequence of surgical segments in the treatment of multi-level lumbar spinal stenosis by UBE technique has a certain impact on the maintenance of smooth flow and clear visual field. The perfusion water enters the operating area from the outlet of the endoscope and circulates in the order of outflow from the operating channel, so that a clear visual field can be maintained. If the observation channel incision is too long, it will affect the water pressure, water circulation sequence and visual field clarity to a certain extent. Therefore, decompression was completed in the sequence of extending the observation channel incision of the previous surgical segment into the working channel incision of the next segment, which could maintain smooth water circulation throughout the operation, ensure clear visual field and facilitate the operation. In addition, attention should be paid to the protection of the upper decompression area during the intraoperative separation of the muscles at the next segment and the completion of the exposure. Since the decompression of the upper segment has been completed, the operation tools should be wary of straying into the spinal canal and causing nerve damage.

A considerable number of scholars believe that continuous infusion of normal saline during the operation is the main advantage to control bleeding, improve the clarity of surgical visual field and prevent infection. However, the use of high-pressure water should be avoided, because high-pressure irrigation may lead to increased intracranial pressure and postoperative headache [16], and some scholars believe that continuous saline irrigation has no significant correlation with inflammatory environment [17]. Ju-Eun et al. [18] pointed out that there are no high-quality randomized controlled studies or systematic review reports on the infection rate between routine open spinal surgery and UBE. So some scholars have suggested that one of the advantages of the UBE technique is that compared to traditional open spinal surgery, it uses saline continuous irrigation. In our study of 26 patients, there was not a single patient with surgical area infection, and we considered that a large amount of normal saline washed away a large number of harmful bacteria and other sources of infection, keeping the surgical area clean, and there was 1 subcutaneous hematoma, which was considered to be caused by soft tissue extravasation. Therefore, we speculate that this may be the main reason why UBE is generally lower than other techniques in the postoperative spine infection rate.

Decompression surgery is a common surgical method in spinal surgery, but dural tear is a common problem worthy of attention regardless of any method of decompression surgery. Some studies indicated that the incidence of dural tears in the UBE group (3.6%) was lower than that in the microendoscopic lumbar decompression group (8.3%) [19] and the open laminectomy group (18%) [20]. Lee et al. [21] believed that due to the existence of ligaments between the dural membrane and the lamina, the yellow ligament and the dural membrane were attached together, and the operator was prone to tear the dural membrane when separating the space between the yellow ligament and the dural membrane and removing the yellow ligament. Therefore, soft plates can be used to protect the removal of the yellow ligament [22]. Although dural injury is common, especially in the decompression process of multi-level lumbar spinal stenosis, dural tear may be relatively high, but in this study, there was a postoperative dural rupture in 1 patient, and the scope of the rupture was about 5 mm, which may be caused by the intraoperative stripping of nerve tissue and pressure, which may be related to excessive radiofrequency heat, or the longer duration of the patient’s disease. Park JH [23] found in his study that dural rupture might be related to the long-term compression of nerve structure, resulting in related complications. The most common complication of UBE is dural tear, with an incidence of about 1.5–5.8% [24]. In short, the dural tear caused by UBE is multifaceted. Risk factors for dural tear include dura injury caused by instruments or radiofrequency, tissue adhesion, and blurred visual field [25]. Studies suggest that absolute bed rest and simple observation are recommended for dural tears with a range of less than 4 mm [26], while open repair is considered a safe treatment for dural tears with a range of more than 10 mm [27]. However, endoscopic dural repair mostly tests the clinical techniques of surgeons and requires further research and development.

Limitations of this study include the lack of lumbar dynamic radiography to reflect postoperative spinal instability and the lack of lumbar MR imaging to reflect postoperative decompression. Therefore, it is necessary to increase the sample size and data follow-up in future studies.

## Conclusions

In summary, this study believes that UBE is an emerging minimally invasive technique, which can be applied not only to the treatment of single-level lumbar spinal stenosis, but also to multi-level lumbar spinal stenosis, with advantages such as small incision, flexible operation, less trauma, and clear surgical field of view. This surgical method greatly shortened the length of hospital stay, the patients recovered quickly after surgery, and the short-term effect was remarkable.

## Data Availability

The data and materials are available from the medical records department of the Orthopedic Hospital of Xingtai. The datasets used and analyzed during the current study are available from the corresponding author on reasonable request.

## Abbreviations

UBE: Unilateral biportal endoscopy
VAS: Visual analog score
ODI: Oswestry Disability Index
LSD: Least significant difference
ANOVA: Analysis of variance
